# Hooded Vultures *Necrosyrtes monachus* are at risk of extinction in Benin: A result of poaching for belief‐based use and decreasing food availability

**DOI:** 10.1002/ece3.11184

**Published:** 2024-04-01

**Authors:** Clément Daboné, Jacques Boco Adjakpa, Mathias Fanou Dansi, Lindy J. Thompson, Florent Essin Dissou, Peter D. M. Weesie

**Affiliations:** ^1^ Animal Biology and Ecology University Centre of Tenkodogo/University Thomas Sankara Tenkodogo Burkina Faso; ^2^ Animal Biology and Ecology University Joseph Ki‐Zerbo Ouagadougou Burkina Faso; ^3^ Applied Biology University of Abomey‐Calavi Abomey‐Calavi Benin; ^4^ Southern African Wildlife College Kempiana Nature Reserve Hoedspruit South Africa; ^5^ Centre for Functional Biodiversity, School of Life Sciences University of KwaZulu‐Natal Scottsville South Africa; ^6^ Integrated Research on Energy, Environment and Society University of Groningen Groningen The Netherlands

**Keywords:** Benin, decreasing food availability, Hooded Vulture, poaching, risk of extinction, West Africa

## Abstract

In recent years, Hooded Vulture populations in West Africa have decreased substantially. However, in some areas within this region, the species is still relatively abundant. To find out more about the situation in West Africa, we assessed the status of Hooded Vultures in Benin, one of the countries where their status is not well known. We conducted road counts on paved and unpaved roads and along small trails over a total of 1451 km. We also conducted interviews with local abattoir watchmen, veterinarians, butchers and foresters to examine potential threats to this species. A total of 52 Hooded Vultures were counted mostly in the departments of Atacora (32) but also in Alibori (10) and Borgou (10). The relative abundance was four Hooded Vultures per 100 km, highlighting the near extirpation of this bird from Benin. A total of 49 interviews revealed that poaching for belief‐based use (through shooting and traps) and decreasing food availability remain the most important threats for Hooded Vultures in northern Benin. If these threats are not mitigated, we predict the extirpation of the Hooded Vulture outside protected areas in Benin within the next two decades, possibly even sooner. Conservation measures, including awareness campaigns, and the improvement and enforcement of environmental legislation, must be urgently implemented to improve the protection of this Critically Endangered vulture species.

## Graphical abstract

In recent years, Hooded Vulture populations in West Africa have decreased substantially. To find out more about the situation in West Africa, we assessed the status of Hooded Vultures in Benin, one of the countries where their status is not well known. The results highlighted the near extirpation of this bird from Benin and poaching for belief‐based use and decreasing food availability were the most important threats for this species.

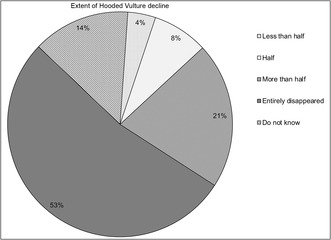

## 1 INTRODUCTION

In recent decades, the catastrophic decline in African vulture populations has been of particular concern (Gbogbo & Awotwe‐Pratt, 2008; Mullié et al., 2017; Thiollay, 2006a, 2006b). However, there have been relatively few studies on the populations trends of vultures in West African countries (Mullié et al., 2017; Ogada et al., 2016; Ogada & Buij, 2011; Rondeau & Thiollay, 2004; Thiollay, 2006a, 2006b). Large‐scale vulture counts (conducted along roads and in formally protected areas) in Mali, Burkina Faso and Niger found vulture populations in severe decline and confined largely to protected areas, with the Hooded Vulture the only species that is still widespread and seen close to human settlements (Thiollay, 2006a, 2006b, 2007a, 2007b). From 1970s to 2004, the number of Hooded Vultures in West Africa declined from 84 individuals every 100 km to 46, a decrease of 45% (Thiollay, 2006a, 2006b). Likewise, Mullié et al. (2017) found a decline of 85% in the number of Hooded Vultures in Dakar, Senegal, over 50 years (1969–2016). A severe decrease in Hooded Vulture numbers was also recorded in Edo State, southern Nigeria (Nosazeogie et al., 2018). Consequently, in 2015, the status of the Hooded Vulture was uplisted to Critically Endangered on the IUCN Red List of Threatened Species (BirdLife International, 2017).

In contrast, in central Burkina Faso, Banjul, the coastal and Western regions of The Gambia, the Ziguinchor department of southwestern Senegal, Guinea‐Bissau and the southern region of Ghana, Hooded Vultures are reportedly still abundant (Annorbah & Holbech, 2012; Barlow, 2012; Barlow & Fulford, 2013; Gbogbo & Awotwe‐Pratt, 2008; Henriques et al., 2017; Jallow et al., 2016, 2022; Mullié et al., 2017; Smalley, 2016; Thiollay, 2006b). This gave rise to a discussion forum about possibly downlisting the status of the Hooded Vulture to Endangered on the IUCN Red List of Threatened Species (D. Ogada, pers. com.; BirdLife International, 2022), although the decision was made to maintain the species' status as Critically Endangered.

An assessment of Hooded Vulture population trends in additional West African countries is critical, before re‐considering the possible downlisting of this species in the IUCN Red List. There is also a need to increase our knowledge on the threats to this species in West Africa, as highlighted in the “Multi‐Species Action Plan to Conserve African‐Eurasian Vultures” (Botha et al., 2017). In this study, we use road counts to explore the status of Hooded Vultures outside of protected areas, in northern Benin. With the high demand for vulture body parts in Benin and neighbouring Nigeria and the increasing profitability of the regional trade in vulture body parts (Buij et al., 2016; Daboné et al., 2023; Nikolaus, 2011), we expected that Hooded Vulture would have become rare, with poaching for belief‐based use being the main threat to Hooded Vultures in this area.

## 2 METHODS

### 2.1 Study area

**FIGURE 1:**
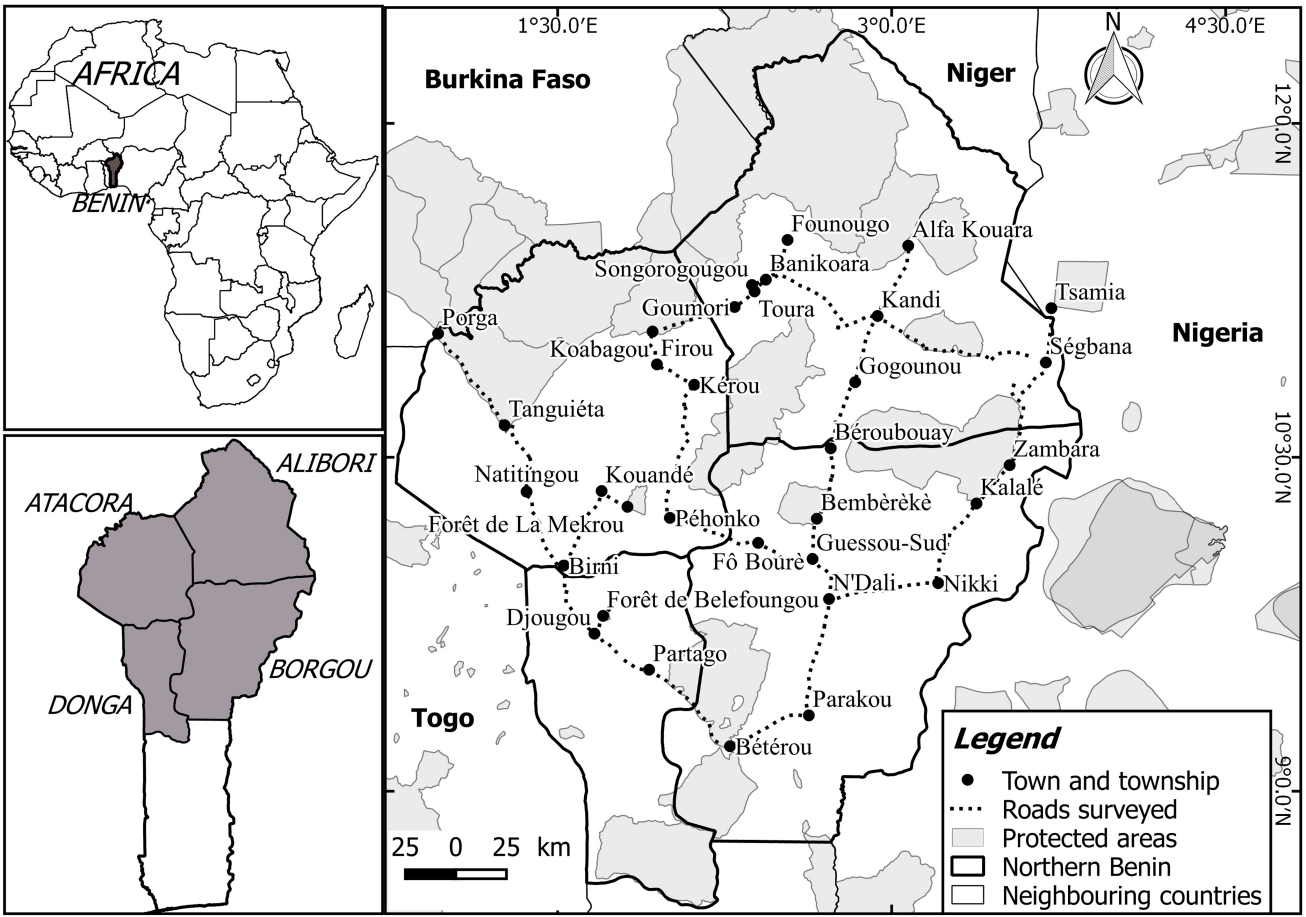
The study area and roads surveyed showing towns, townships and protected areas in northern Benin in 2019.

The Republic of Benin is situated in West Africa between the latitudes 6°10′ N and 12°25′ N and longitudes 0°45′ E and 3°55′ E. Our area of interest in this study was the northern part of the country, particularly the departments of Atacora, Alibori, Borgou and Donga (Figure 1), where Hooded Vultures were formerly recorded (Claffey, 1995; Dowsett, 1993; Dowsett & Forbes‐Watson, 1993; Green & Sayer, 1979). It covers a land area of 83,723 km^2^ and has an estimated population at 3397104 inhabitants (Institut National de la Statistique et de l'Analyse Economique, 2013). The mean annual rainfall varies from 900 to 1300 mm and the mean annual temperature ranges from 26 to 28°C and it may occasionally reach 35–40°C in localities such as Kandi and Malanville (Adomou, 2005). Two climate zones can broadly be distinguished: the northern zone is characterized by a truly Sudanian climate while the transition zone is subhumid or sub‐Sudanian (Adjanohoun et al., 1989; Akoègninou, 2004). Two major vegetation zones can be distinguished in northern Benin: the zone of undifferentiated Sudanian woodland and the zone of Sudanian woodland (Natta, 2003; White, 1983).

### 2.2 Population assessment

To assess Hooded Vulture abundance, road counts were conducted on 20 days from 30 January to 18 February 2019 in the early dry season. We chose this method because it enables large areas to be covered relatively quickly, and large numbers of individuals and species (including low‐density species) can be recorded (Thiollay, 2006b). Counts were done along paved and unpaved roads and on small trails, divided into 15 transects. These transects had a total length of 1451 km, and a mean (± SD) length of 97 ± 25.7 km; range: 48–149 km (Figure 1, Table 1).

**TABLE 1:** Transects used in Hooded Vulture road counts in Northern Benin.

No.	Code	Transect itinerary	Number of times driven	Length (km)
1	PB	Porga (Burkina Faso border) – Tanguiéta – Natitingou – Birni (PB_1_ = 57; PB_2_ = 52; PB_3_ = 40)	PB (once)	149
2	BM	Birni – Kouandé – Forêt de La Mekrou (BM_1_ = 46; BM_2_ = 18)	BM (twice)	64
3	BB	Birni – Djougou – Forêt de Belefoungou (BB_1_ = 38; BB_2_ = 10)	BB_1_ (once); BB_2_ (twice)	48
4	DB	Djougou – Partago – Bétérou (DB_1_ = 34; DB_2_ = 59)	DB (once)	93
5	BG	Bétérou – Parakou – N'Dali – Guessou‐Sud (BG_1_ = 43; BG_2_ = 62; BG_3_ = 25)	BNB (once)	130
6	GG	Guessou‐Sud – Bembéréké – Béroubouay – Gogounou (GG_1_ = 22; GG_2_ = 39; BD_2_ = 41)	BG (once)	102
7	GA	Gogounou – Kandi – Alfa Kouara (Park W Benin) (GA_1_ = 65; GA_2_ = 40)	GA (once)	105
8	KF	Kandi – Banikoara – Founougo (KF_1_ = 71; KF_2_ = 25)	KF_1_ (once); KF_2_ (twice)	96
9	BK	Banikoara (Songorogougou, Toura) – Goumori – Koabagou (Pendjari National Park) (BK_1_ = 33; BK_2_ = 55)	BK (once)	88
10	KP	Koabagou – Firou – Kérou – Péhonko (KP_1_ = 21; KP_2_ = 21; KP_3_ = 74)	KP (once)	116
11	PG	Péhonko – Fô Bourè – Guessou‐Sud (PG_1_ = 49; PG_2_ = 39)	PG (once)	88
12	GN	Guessou‐Sud – N'Dali – Nikki (GN_1_ = 25; GN_2_ = 54)	PN (once)	79
13	NS	Nikki – Kalalé – Zambara (NZ_1_ = 44; NZ_2_ = 30)	NS (once)	74
14	NS	Zambara – Ségbana – Tsamia (ZT_1_ = 75; ZT_2_ = 25)	NS (once)	100
15	TK	Tsamia (Nigeria) – Ségbana – Kandi (TK_1_ = 25; TK_2_ = 94)	TK_1_ (twice); TK_2_ (once)	119
Total				1451

*Note*: Distances (km) between successive towns/townships are given in brackets.

Transects were chosen according to a nonprobability (purposeful) sampling method, which involved selecting routes where the probability of encountering a species is high (Patton, 2002). Given that Hooded Vultures have a close commensal relationship with people in West Africa (Daboné et al., 2019, 2022; Mundy et al., 1992), we chose transects that linked the 33 main towns and townships that are reported to be potential strongholds for Hooded Vultures in northern Benin (Claffey, 1995; Dowsett & Forbes‐Watson, 1993; Green & Sayer, 1979; Clément Daboné pers. obs.). Furthermore, as Hooded Vultures are commensal in West Africa, even in protected areas (Thiollay, 2006b), we included routes along the edges of national parks and classified forests, whenever possible. In addition, haphazard records tend to offset each other over long distances (Thiollay, 2006b). Therefore, to maximize the likelihood of detecting this species, while keeping within the same departments and the same dominant habitat between several towns (Thiollay, 2006b), we took care that each transect should include only three or four towns/townships over a distance no longer than 150 km.

All counts were performed by car, driving slowly with frequent stops, in fine weather, between sunrise and sunset, except during the hot early afternoon hours when Hooded Vulture are less likely to be active (Thiollay, 2006a). All journeys were made from 07:00 to 11:00 in the morning and from 14:00 to 17:00 in the afternoon. Surveillance was made from the front passenger seat and the rear seat of the moving car using binoculars (Nikon Prostaff 3S 10 × 42). All Hooded Vultures seen perched or flying, on either side of the road, were counted. Two observers (the first author and another experienced observer) looked carefully for Hooded Vultures while driving slowly (<40 km/h, but slower (20 km/h) in towns and at the edge of the parks), and with frequent stops to identify distant birds or scan the landscape (Thiollay, 2006b). The length of transects was measured using the vehicle odometer and road maps (Barlow & Fulford, 2013; Henriques et al., 2018; Thiollay, 2006b).

### 2.3 Exploring threats to Hooded Vultures

To examine threats to Hooded Vultures in northern Benin, a survey was carried out concurrent to the road counts. The nonprobability (purposeful) sampling method (Patton, 2002) was used to identify individuals (or groups of individuals) who were particularly knowledgeable about or experienced with Hooded Vultures (Cresswell & Plano Clark, 2011). The people we targeted were abattoir watchmen, veterinarians, butchers and foresters, all of whom have a close relationship with this species (Daboné et al., 2022, 2023; Mundy et al., 1992; Thiollay, 2006b; Weesie & Belemsobgo, 1997) and accordingly are key stakeholders for its conservation in West Africa.

When carrying out the road census, we stopped (for about 15–20 min) in the local abattoirs, if there were any, whenever we reached towns and townships. There, we collected data using unstructured interviews with abattoir watchmen, veterinarians and butchers who we found on the site and who were willing and available to participate. Interviews took an average of 7 min to complete (range: 5–12 min). This process was complemented by direct observation to search for any indications of the persecution of Hooded Vultures. Moreover, once in a locality, if we had enough time (e.g., when we stopped a census from 11:00 to 14:00, or if we had to spend the night), then we completed an unstructured interview with one forester (the Head of Service or their representative). In Benin, foresters are assigned to control and safeguard protected species including vultures. So, they were considered, in this study, as those who would provide informative, high‐quality responses to our survey questions, and therefore, their surveys took substantially longer, between 10 and 30 min. We obtained informed consent from all interviewees, and we also assured them that their participation was voluntary, that they could terminate the interview at any time, and that we would ensure their anonymity. Photographs of Hooded Vultures were used to aid the respondents' identification of the species.

We began each interview by assessing whether interviewees know Hooded Vultures and whether they have seen them in their area of residence. We then asked interviewees whether they were aware of the Hooded Vulture population decline and if they could give reasons for the decline. Finally, we asked interviewees to tell us what else they know about Hooded Vultures.

### 2.4 Data analyses

Using data from our road counts, we calculated Hooded Vulture encounter rates for each transect (expressed as number of birds per 100 km of transect), to show the relative abundance of this species in northern Benin. One entire transect and three partial transects were counted twice on the same day, but at different times (i.e., during return travels). The maximum number of Hooded Vultures recorded in either count was then retained.

Interview statements were read, re‐read and classified based on their similarities, dissimilarities, key words and phrases (Babbie, 1999). This led to the identification of key themes and relationships from the data (Burnard et al., 2008; O'Connor & Gibson, 2003). Our personal observations of signs of persecution of Hooded Vultures provided supplementary information, which complemented our interview data. Data from unstructured interviews were summarized using descriptive statistics in tables and pie charts.

## 3 RESULTS

### 3.1 Abundance of Hooded Vultures

Over a cumulative total of 1451 km driven over all 15 transects, a total of 52 Hooded Vultures were counted, mostly in the departments of Atacora (32) but also in Alibori (10) and Borgou (10). There were no sightings of these birds in the department of Donga (Figure 1). The mean (± SD) count per transect was 3.46 ± 6.16 (Table 2). Counts ranged from zero (in 10 transects out of 15) to a maximum of 20 Hooded Vultures on “Transect 10” from Koabagou, through Firou, Kérou, to Péhonko (Table 2). Hooded Vultures were encountered very rarely, only five times over 20 days of fieldwork. The relative abundance (mean number of Hooded Vulture recorded per 100 km over all 15 transects counts) was 3.58 (± 5.70), highlighting the near‐disappearance of this bird in Benin.

**TABLE 2:** Numbers of Hooded Vultures (HV) recorded per transect, and mean numbers of Hooded Vultures recorded per 100 km, for all 15 transects used in northern Benin.

Code	Transect itinerary	Length (km)	HV per transect	Mean HV per 100 km
PB	Porga (Burkina Faso border) – **Tanguiéta** (5) – Natitingou (7) – Birni	149	12	8.05
BM	Birni – Kouandé – Forêt de La Mekrou	64	0	0
BB	Birni – Djougou – Forêt de Belefoungou	48	0	0
DB	Djougou – Partago – Bétérou	93	0	0
BG	Bétérou – Parakou – N'Dali – Guessou‐Sud	130	0	0
GG	Guessou‐Sud – Bembéréké – Béroubouay – Gogounou	102	0	0
GA	Gogounou – Kandi – Alfa Kouara (Park W Benin)	105	0	0
KF	Kandi – Banikoara – **Founougo** (8)	96	8	8.33
BK	**Banikoara** (2) – Goumori – Koabagou (Pendjari National Park)	88	2	2.27
KP	Koabagou – Firou – Kérou – **Péhonko** (20)	116	20	17.24
PG	Péhonko – Fô Bourè – Guessou‐Sud	88	0	0
GN	Guessou‐Sud – N'Dali – Nikki	79	0	0
NS	Nikki – Kalalé – **Zambara** (10)	74	10	13.51
NS	Zambara – Ségbana – Tsamia	100	0	0
TK	Tsamia (Nigeria) – Ségbana – Kandi	119	0	0
Total		1451	52	3.58

*Note*: For each transect, the number of Hooded Vultures encountered is given in brackets, and the localities of these sighting are shown in bold text.

### 3.2 Assessment of threats

#### 3.2.1 Knowledge of Hooded Vultures

**FIGURE 2:**
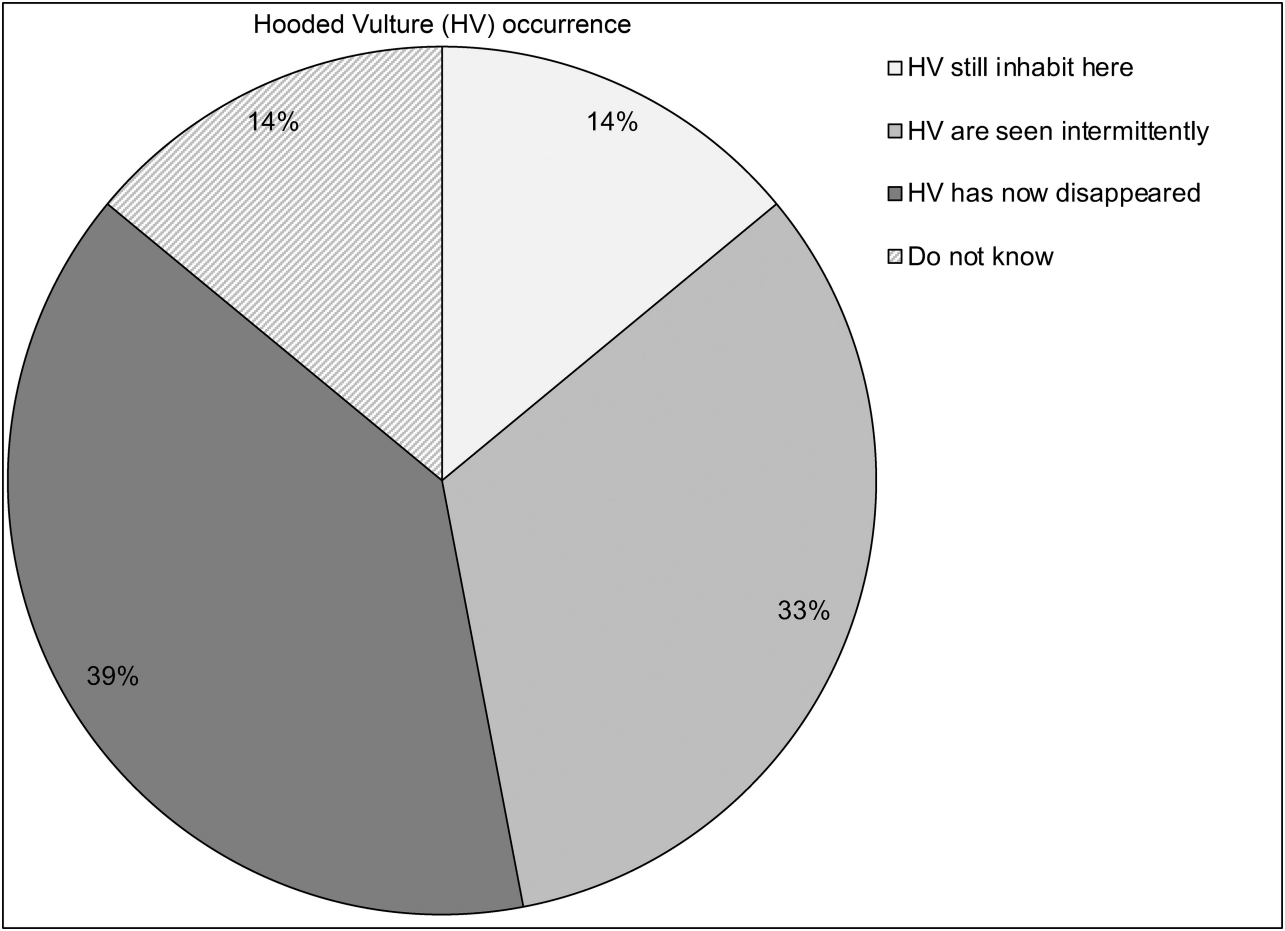
Knowledge and perceptions of the occurrence of Hooded Vultures (HV).

In the 33 localities that the transects passed through, we completed a total of 49 interviews with 22 butchers, 12 foresters, 8 veterinarians and 7 abattoir watchmen. All interviewees were men, over 30 years old. The survey revealed that 46 respondents (94%) were confident that they knew the Hooded Vulture, according to the photos presented to them. Only seven (14%) reported that Hooded Vultures still inhabit their town or township, suggesting the near‐disappearance of this bird in northern Benin. Indeed, 19 interviewees (39%) stated that Hooded Vultures once existed in their locality but have now disappeared, 16 (33%) reported that Hooded Vultures are observed intermittently, while seven (14%) reported being unaware of Hooded Vulture presence in their town or township (Figure 2).

#### 3.2.2 Perceptions of the Hooded Vulture population trend, and the extent and cause of its decline

**FIGURE 3:**
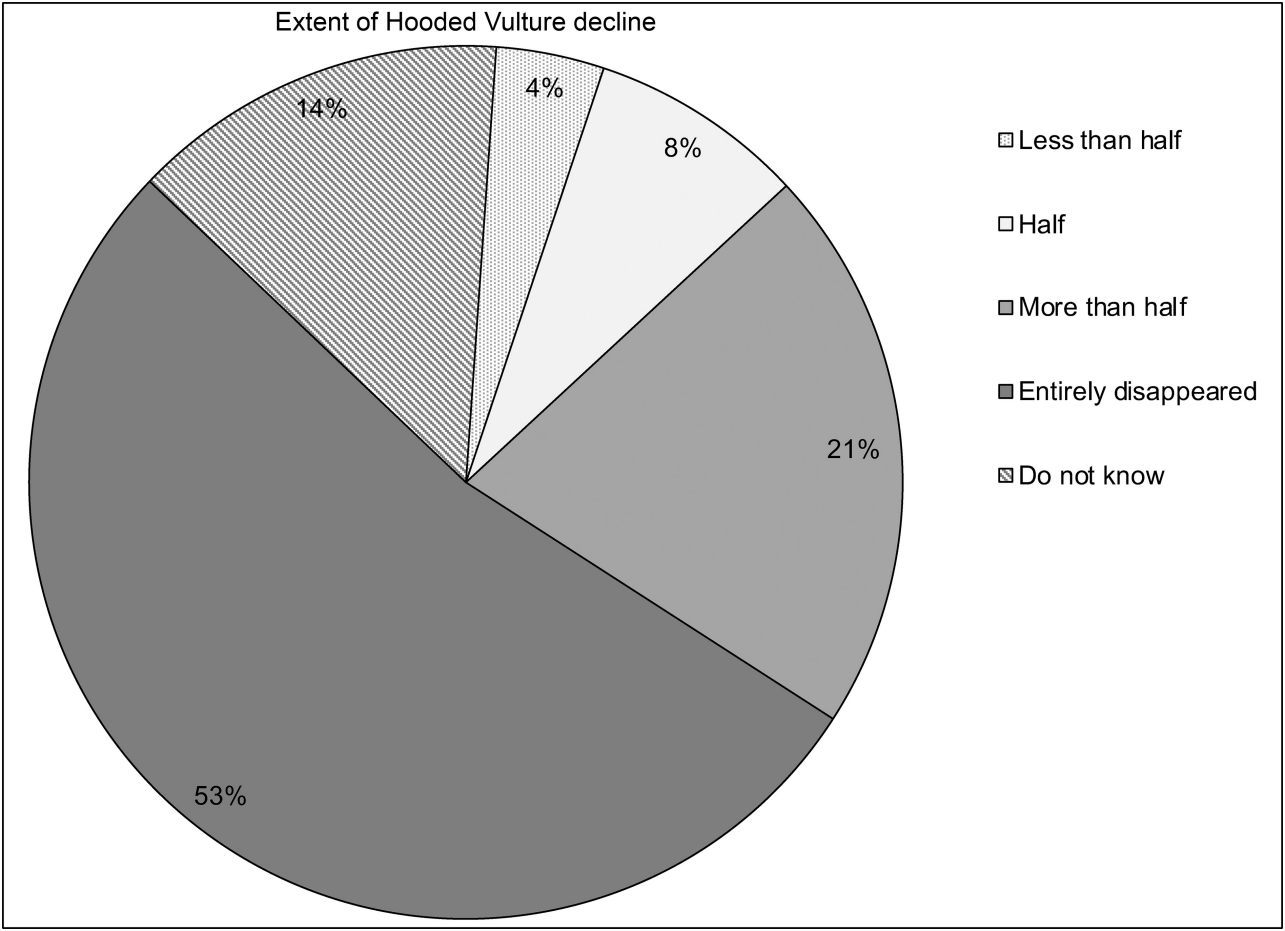
Knowledge of the extent of the decline in Hooded Vulture population size.

**FIGURE 4:**
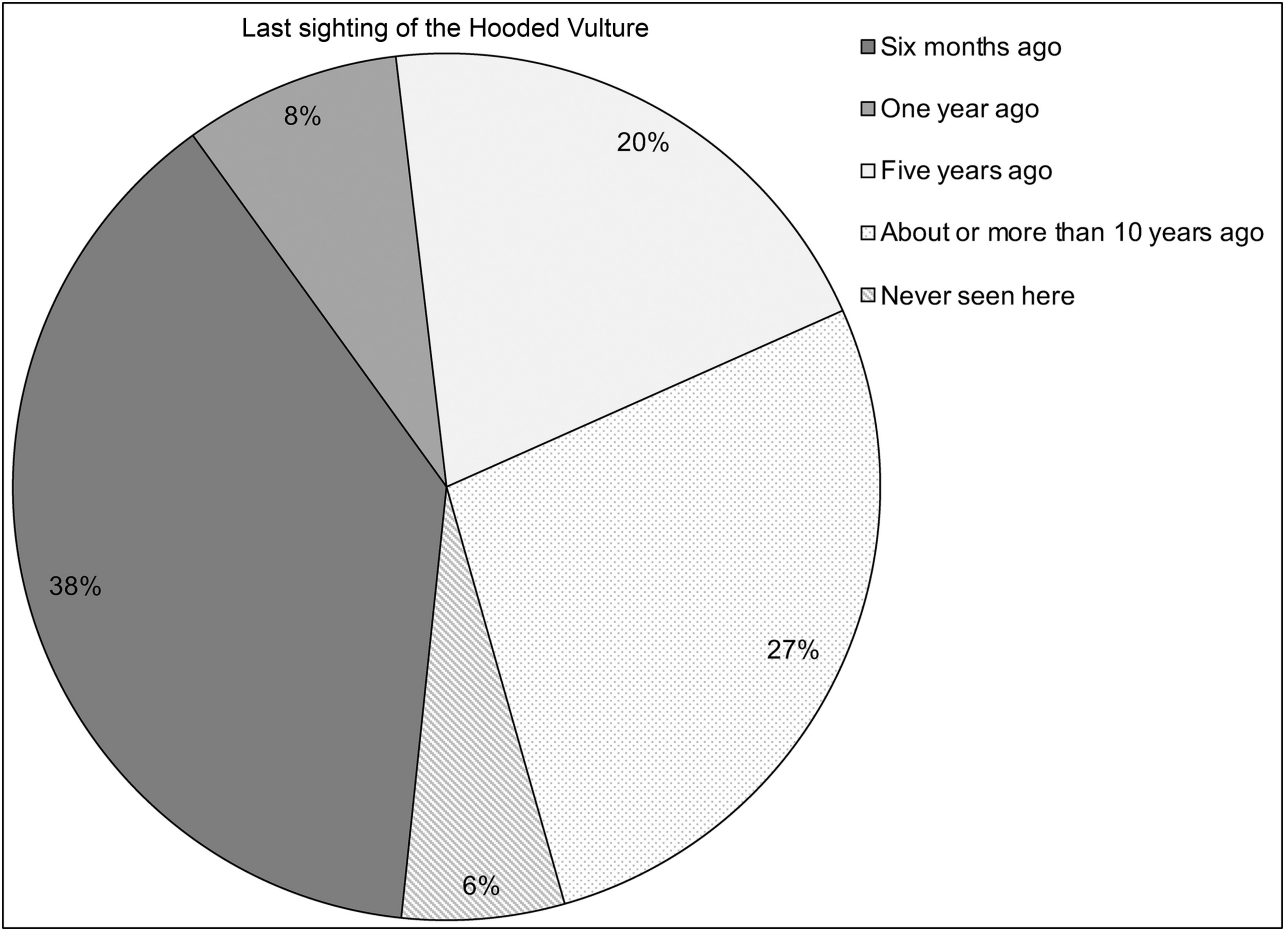
Last sighting of the Hooded Vulture according to interviewees.

**FIGURE 5:**
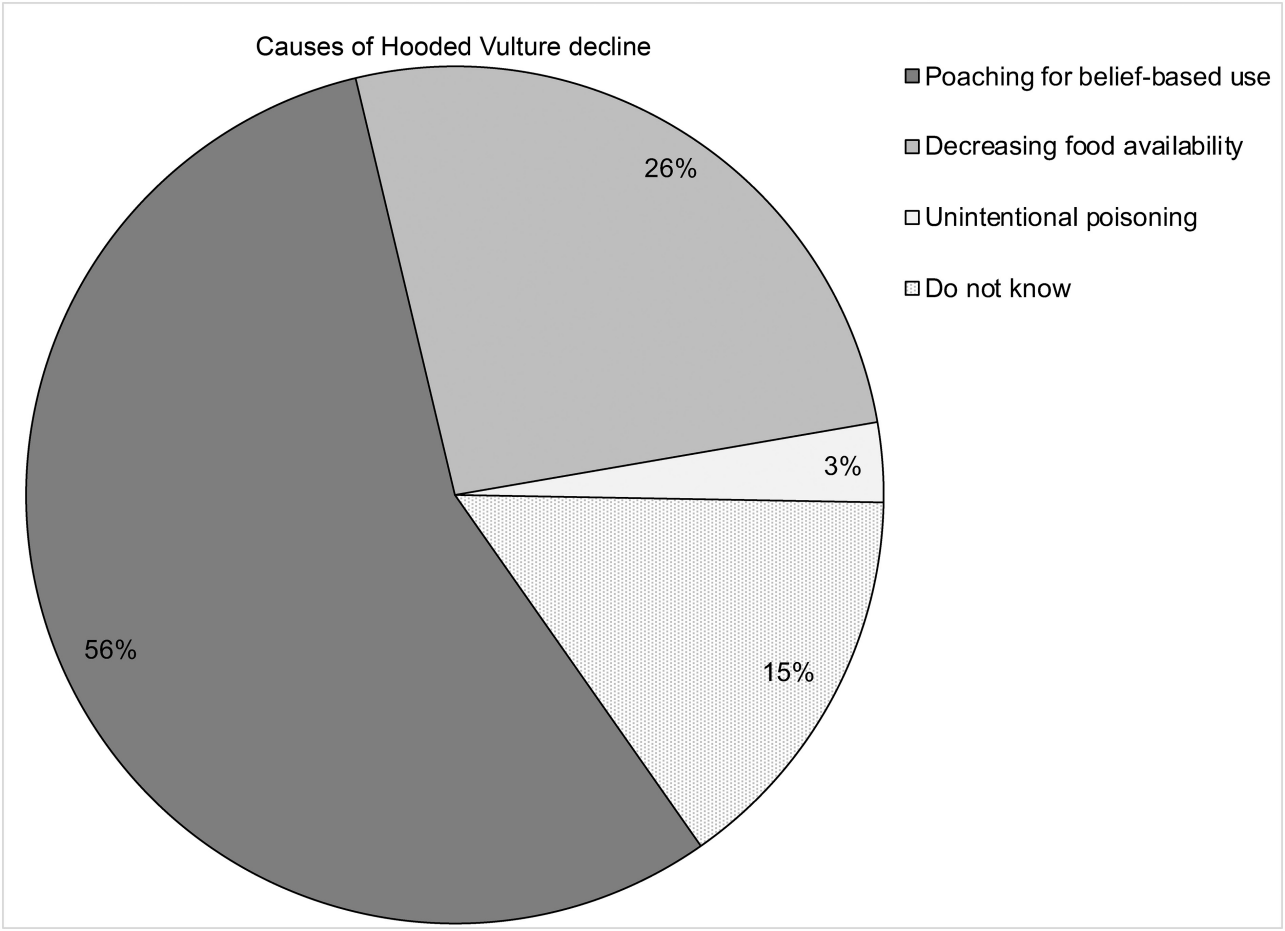
Knowledge about the causes of Hooded Vulture declines.

The vast majority of respondents (92%, *n* = 45, Tables A1 and A2 in Appendix) had noticed a decrease in the Hooded Vulture population. Overall, 53% (*n* = 26) reported that Hooded Vultures have already disappeared entirely from their locality. About 20% of respondents (*n* = 10) thought the population had decreased by more than half, while 8% (*n* = 4) thought it had decreased by half (Figure 3). In accordance with these statements, about 47% (*n* = 23) of the respondents highlighted that Hooded Vultures were last seen in their locality over 5 years ago (Figure 4). They suggested that the main reasons for the disappearance of Hooded Vultures in their town or township include poaching (mostly by shooting or trapping) for belief‐based use (suggested by 56% of respondents), decreasing food availability (26% of respondents) and unintentional poisoning (3% of respondents) (Figure 5). In line with these statements, we personally saw traps for Hooded Vulture at three abattoirs, and a hunter holding a shotgun, waiting for Hooded Vultures, at one abattoir (Clément Daboné and Florent Essin Dissou pers. obs.). Furthermore, we did not see any animal carcasses or organs (unsuitable for human consumption) on the ground at abattoirs or in the surrounding areas. Most concerning, we repeatedly saw people at some abattoirs keeping organs that had been declared unsuitable for human consumption by veterinary services (i.e., liver, heart, lungs and even fetuses pulled from the pregnant females) and these could have been offered as food to vultures.

#### 3.2.3 Other statements about Hooded Vultures

Our interviewees reported some cultural uses for Hooded Vultures; 43% said that Hooded Vultures are used to bring good luck when gambling, trading, and in competitions and contests, 16% said that Hooded Vulture body parts are used to cure a range of illnesses or prevent misfortune, and 14% of our respondents said that these birds are used to cast evil curses or spells (Table 3). They also reported some observations on the vultures' behaviour; 18% of respondents said that foresters, rangers and eco‐guards use Hooded Vulture behaviour to track poacher activity or to find animal mortalities in protected areas, and 8% said that Hooded Vulture usually follows livestock breeders in search of food (Table 3).

**TABLE 3:** Other statements reported about Hooded Vultures and their threats, including the number of respondents within abattoir watchmen (*n*_1_), veterinarians (*n*_2_), foresters (*n*_3_) and butchers (*n*_4_).

Statement about Hooded Vultures	Number (*n*) and percent (%) of respondents who agreed with each statement				
	Abattoir watchmen	Veterinarians	Foresters	Butchers	Total
	*n*_1_ (%)	*n*_2_ (%)	*n*_3_ (%)	*n*_4_ (%)	*n* (%)
Hooded Vultures are used to cast evil curses or spells	1 (14.3)	3 (37.5)	1 (8.3)	2 (9.9)	7 (14.3)
Hooded Vulture body parts are used to cure a range of illnesses or prevent misfortune	2 (28.6)	1 (12.5)	2 (16.7)	3 (13.6)	8 (16.3)
Foresters, rangers and eco‐guards use Hooded Vulture behaviour to track poacher activity or to survey animal mortalities in protected areas	0 (0)	2 (25.0)	5 (41.7)	2 (9.1)	9 (18.4)
Hooded Vultures are used to bring good luck when gambling, trading, and in competitions and contests	4 (57.1)	1 (12.5)	4 (33.3)	12 (54.5)	21 (42.9)
Hooded Vultures usually follow livestock breeders in search of food	0 (0)	1 (12.5)	0 (0)	3 (13.6)	4 (8.2)

*Note*: The total number of respondents (*n*) is shown, along with the number of respondents expressed as a percentage of the total (%).

## 4 DISCUSSION

### 4.1 Abundance of Hooded Vultures

This study confirms that the Hooded Vulture population has declined significantly across northern Benin, the only part of the country where this bird was formerly recorded (Claffey, 1995; Dowsett & Forbes‐Watson, 1993; Green & Sayer, 1979). We encountered a relatively high abundance of Hooded Vultures in the Atacora department with about 62% of individuals (*n* = 52) recorded here. The reason for their relative abundance in this department is probably the proximity to the Pendjari National Park a well‐managed site, which obviously forms a critical refuge for this declining vulture (Thiollay, 2006b). Indeed, following the surge in demand for vulture body parts in this area (Daboné et al., 2023; Henriques et al., 2020) with the decreasing food availability in unprotected lands (Ogada & Buij, 2011), Hooded Vulture populations are expected to use protected areas as refugia (Shaw et al., 2024). As such, Shaw et al. (2024) stated that species facing to the broad range of pressures and suffering a sharpest drop in abundance inevitably became more dependent on protected areas than those showing little or no change. Although no quantitative data were available to compare with those in the current study, the population size of this species, assessed at 52 individuals encountered during 20 days of fieldwork, over a total length of 1451 km, was low. This supports the results of Thiollay (2007a) who surveyed part of Pendjari National Park near the Burkina Faso border, and reported declines of 31% for Hooded Vultures in this protected area in northern Benin. In unprotected areas, Claffey (1995) and F. Lemaire and B. Dowsett (data from 2009) in Ogada and Buij (2011), reported that Hooded Vultures were frequently seen year‐round in riparian areas in the Bétérou area. However, in the current study, surveying this area, we did not encounter any Hooded Vultures.

The frequency of Hooded Vultures recorded during road counts in northern Benin was about four individuals per 100 km, which is very low, compared to 291, 255 and 734 individuals every 100 km recorded in recent surveys in Gambia (Barlow & Fulford, 2013; Henriques et al., 2018; Jallow et al., 2022). Interestingly, in neighbouring Burkina Faso, a recent nationwide road count recorded 1365 Hooded Vultures over 2261 km, with an average of 60 individuals per 100 km (Daboné et al., 2024), which suggests there may have been a massive decline in Hooded Vulture populations in Benin. Using a different survey method to ours, a similarly large decline in Hooded Vulture populations was recorded in Edo State, Nigeria, where only 37 Hooded Vultures were counted during 20 count points (at dumpsites, roosting sites and on roads) in 13 study areas (Nosazeogie et al., 2018).

For centuries, Hooded Vultures in West Africa have occurred in large numbers and were strongly commensal with humans (Mundy et al., 1992; Thiollay, 2006b, 2007a). They were frequently observed in public places, at landfill sites, in open dumpsters, and at slaughterhouses and meat markets, often just a few metres away from people (Mundy et al., 1992; Ogada & Buij, 2011; Weesie & Belemsobgo, 1997). Indeed, some studies in recent decades found that Hooded vultures are still abundant in some parts of West Africa, especially in central Burkina Faso, Banjul, the coastal and Western regions of Gambia, the Ziguinchor department of southwestern Senegal, Guinea‐Bissau and the southern region of Ghana (Annorbah & Holbech, 2012; Barlow, 2012; Barlow & Fulford, 2013; Gbogbo & Awotwe‐Pratt, 2008; Henriques et al., 2017; Jallow et al., 2016; Mullié et al., 2017; Smalley, 2016; Thiollay, 2006b). Unfortunately, our results clearly demonstrate that the situation for Hooded Vultures in Benin is critical. Therefore, supported by these road count data, we alert the governments, NGOs, ornithological research institutions and the media to the plight of Hooded Vultures in northern Benin.

Hooded Vultures in northern Benin occur in relatively low numbers, and there is an increasingly profitable regional trade in vulture body parts in this area (Daboné et al., 2023), along with reduced food availability. If these threats are not mitigated, then, on the basis on empirical studies and models that have estimated the median annual rate of decline for this species at 3.3% in this region (Ogada et al., 2016), we predict the extirpation of the Hooded Vulture outside protected areas in Benin in two decades, possibly even sooner.

### 4.2 Assessment of threats

#### 4.2.1 Knowledge of Hooded Vultures

Concurrent to the road counts, we also explored the threats to Hooded Vultures in northern Benin through interviews. Our findings suggest that local people have good knowledge of Hooded Vultures; the species was identified by almost all interviewees, but only a few (14% of respondents) stated that it still occurs in their town or township. This suggests that this species was common in the area, as previously reported (Claffey, 1995; Mundy et al., 1992; Ogada & Buij, 2011), and that its situation has worsened recently. Furthermore, the vast majority of the respondents (92%) had noticed a decrease in the Hooded Vulture population, reinforcing the results obtained from our road counts. Some of the interviewees (12%) stated that Hooded Vultures were last seen in their locality ≥15 years ago, suggesting that the Hooded Vulture population in northern Benin has been declining since at least the 2000s.

#### 4.2.2 Threats to Hooded Vultures

The interviewees suggested that causes of decline were mainly poaching for belief‐based use and decreasing food availability. Previous studies highlighted Nigeria and Benin as important hubs for the trade in vulture body parts in West Africa (Buij et al., 2016; Nikolaus, 2011). Hooded Vultures are killed for this purpose in these countries, and in neighbouring countries (spanning Burkina Faso to Chad) where they occur in greater numbers (Buij et al., 2016; Craig et al., 2018; Daboné et al., 2023; Mander et al., 2007; Nikolaus, 2011). Worryingly, Hooded Vultures are killed using shotguns and trapped with snares (we witnessed this at four abattoirs, CD pers. obs.), and poachers seemingly operate without fear of apprehension by the local forestry service.

In Benin, all vulture species are protected by several legislative decrees of which the most recent is the N° 2011‐394 of 28 May 2011 (République du Bénin, 2011). According to this decree, the hunting, capture, possession and trade of vultures are banned, and any person infringing this provision shall be liable to sentences ranging from 3 months to 3 years and/or to a fine of 100,000 to 500,000 CFA (~ USD 160–830). Nevertheless, while performing fieldwork, we were occasionally mistaken for poachers or traders, and offered vulture carcasses for 60,000–100,000 CFA (~ USD 100–160) each, which is 12–20 times the usual price paid in Burkina Faso (Daboné et al., 2023). Furthermore, we encountered a traditional healer living in Nikki locality with two live Hooded Vultures in a cage, who had declined several offers from Nigerians of up to 300,000 CFA (~ USD 500) for a live Hooded Vulture. He alleged that Hooded Vultures are valuable because he can use their behaviour and communicate with them to treat all kinds of physical, emotional and spiritual illnesses.

Decreasing food availability has been reported as a potential threat to Hooded Vultures in West Africa (Ogada & Buij, 2011). Indeed, this threat appears to be severe for Hooded Vulture in northern Benin; during 20 days of fieldwork, we saw no carcasses or organs (unsuitable for human consumption) left at abattoirs for Hooded Vulture to feed on, as is commonly found throughout Burkina Faso. Instead, we saw people keeping parts of slaughtered animals, although they were not clear or consistent about the purpose for which these parts were harvested. Although we have no firm evidence and rely mostly on hearsay in this regard, we believe that people consume these carcasses or organs that could have been used to feed Hooded Vultures. For this vulture, which is almost completely dependent on anthropogenic food resources in this region (Thiollay, 1977), the reduction in the availability of discarded offal and scraps at abattoirs will hasten its extirpation in urban areas of West Africa.

## 5 CONCLUSION

In some areas in West Africa, Hooded Vultures are reportedly still common and abundant (Barlow & Fulford, 2013; Jallow et al., 2016; Smalley, 2016). However, our study highlighted that the situation for Hooded Vultures in West Africa is critical, with a near‐extinction of this species in some areas, particularly in Benin. In light of this, it appears that Hooded Vultures are severely declining in their West African range, but still survive locally in some restricted areas. Where large densities of vultures still occur, there has been a rapid rise in reports of vultures killed using poisoned baits for trade and belief‐based use (Daboné et al., 2023; Henriques et al., 2020). This situation is alarming and presents a serious conservation challenge for Hooded Vultures in this region. Increased attention must be paid to the overall situation for Hooded Vultures in West Africa, before any further attempts to downlist the conservation status of this bird to Endangered on the IUCN Red List of Threatened Species. It would be foolhardy at best and indecent at worst to downlist its status of Endangered, until we have a complete overview of populations trends and threats in the West African subregion.

## 6 CONSERVATION IMPLICATION

Our results have shown that poaching (with poisoned baits, guns and traps) and decreasing food availability are the most important threats for Hooded Vultures in northern Benin and elsewhere in West Africa (Daboné et al., 2023). It is important that these threats be urgently addressed, and priority actions should include: (a) awareness campaigns focusing on the legal protection of Hooded Vultures, highlighting sentences and fines for any person found to be infringing such legislation; (b) the improvement and enforcement of appropriate policy and legislation to guarantee the safety of Hooded Vultures; (c) awareness campaigns specifically covering the health risks associated with the consumption of meat considered unsuitable for humans, to increase food availability for Hooded Vultures; (d) a stronger commitment, collaboration and coordination between West African governments to ensure sustainable vulture conservation and the development of a legal framework for their protection at a regional level; (e) compiling and enacting national and regional action plans for vulture conservation; and (f) the implementation of vulture feeding stations near abattoir sites, which are attended by guards to prevent poaching to thereby protect vultures, and where veterinarians supply carcasses and organs that is unsuitable for human consumption to increase the availability of food for Hooded Vultures, to assist in restoring urban Hooded Vulture populations in northern Benin.

## AUTHOR CONTRIBUTIONS

**Clément Daboné:** Conceptualization (lead); investigation (lead); methodology (lead); writing – original draft (lead). **Jacques Boco Adjakpa:** Project administration (equal); supervision (equal); writing – review and editing (equal). **Mathias Fanou Dansi:** Investigation (supporting); methodology (supporting); resources (supporting). **Lindy J. Thompson:** Data curation (supporting); formal analysis (supporting); writing – review and editing (equal). **Florent Essin Dissou:** Investigation (supporting); methodology (supporting). **Peter D. M. Weesie:** Conceptualization (supporting); funding acquisition (lead); supervision (supporting); validation (supporting).

## FUNDING INFORMATION

This work was supported by the Integrated Research on Energy, Environment and Society (IREES), Faculty of Science and Engineering, University of Groningen, The Netherlands. The authors declare that no funds, grants or other support were received during the preparation of this manuscript.

## CONFLICT OF INTEREST STATEMENT

The authors declare no conflicts of interest.

## Supporting information


Tables S1

## Data Availability

The data gathered (on road counts and surveys) are publicly available at Zenodo data repository (https://doi.org/10.5281/zenodo.8119059).

## Appendix

**TABLE A1:** Knowledge reported about Hooded Vultures and their threats, including the number of respondents within abattoir watchmen (*n*_1_), veterinarians (*n*_2_), foresters (*n*_3_) and butchers (*n*_4_).

	Extent of agreement by respondent				
	Abattoir watchmen	Veterinarians	Foresters	Butchers	Total
	*n*_1_ (%)	*n*_2_ (%)	*n*_3_ (%)	*n*_4_ (%)	*n* (%)
Knowledge of Hooded Vulture					
Interviewee knows Hooded Vultures (according to the photos presented to them)	7 (100)	7 (87.5)	12 (100)	20 (90.9)	46 (93.9)
Interviewee does not know Hooded Vultures (according to the photos presented to them)	0 (0)	1 (12.5)	0 (0)	2 (9.1)	3 (6.1)
Hooded Vulture presence in their locality					
Hooded Vultures still occur here	3 (42.9)	0 (0)	0 (0)	4 (18.2)	7 (14.3)
Hooded Vultures are seen intermittently in this area	1 (14.2)	3 (37,5)	5 (41.7)	7 (31.8)	16 (32.6)
Hooded Vultures previously occurred in this locality but have now disappeared	3 (42.9)	2 (25)	5 (41.7)	9 (40.9)	19 (38.8)
Hooded Vultures never occurred in this area	0 (0)	0 (0)	0 (0)	0 (0)	0 (0)
Do not know	0 (0)	3 (37.5)	2 (16.6)	2 (9.1)	7 (14.3)
Hooded Vulture population trends					
Numbers of Hooded Vultures are decreasing	7 (100)	7 (87.5)	12 (100)	19 (86.3)	45 (92)
Numbers of Hooded Vulture are stable or increasing	0 (0)	0 (0)	0 (0)	0 (0)	0 (0)
Do not know	0 (0)	1 (12.5)	0 (0)	3 (13.6)	4 (8)
Extent of Hooded Vulture declines					
Hooded Vulture numbers have decreased by less than half	0 (0)	0 (0)	0 (0)	2 (9.1)	2 (4.1)
Hooded Vultures have decreased by half	0 (0)	1 (12.5)	1 (8.3)	2 (9.1)	4 (8.2)
Hooded Vultures have decreased by more than half	3 (42.8)	0 (00)	1 (8.3)	6 (27.3)	10 (20.4)
Hooded Vultures have already disappeared entirely here	3 (42.8)	4 (50.0)	9 (75.0)	10 (45.4)	26 (53.0)
Do not know	1 (14.2)	3 (37.5)	1 (8.3)	2 (9.1)	7 (14.3)
Where Hooded Vulture are encountered today in Benin					
Hooded Vultures are encountered here	3 (42.8)	1 (12.5)	2 (16.7)	5 (22.7)	11 (22.5)
Hooded Vultures are encountered only in protected areas	3 (42.8)	4 (50.0)	9 (75.0)	7 (31.8)	23 (46.9)
Do not know	1 (14.3)	3 (37.5)	1 (8.3)	10 (45.5)	15 (30.6)
Last sighting of Hooded Vultures in their locality					
Less than 1 month ago	3 (42.8)	2 (25.0)	1 (8.3)	5 (22.7)	11 (22.4)
6 months ago	0 (0)	0 (0)	4 (33.3)	4 (18.2)	8 (16.3)
1 year ago	1 (14.3)	0 (0)	1 (8.3)	2 (9.1)	4 (8.2)
5 years ago	1 (14.3)	2 (25.0)	4 (33.3)	3 (13.6)	10 (20.4)
10 years ago	1 (14.3)	1 (12.5)	0 (0)	5 (22.7)	7 (14.3)
15 years ago	0 (0)	1 (12.5)	2 (16.7)	1 (4.5)	4 (8.2)
At least 20 years ago	1 (14.3)	1 (12.5)	0 (0)	0 (0)	2 (4.1)
Never seen here	0 (0)	1 (12.5)	0 (0)	2 (9.1)	3 (6.1)
Causes of Hooded Vulture declines					
Poaching for belief‐based use	7 (63.6)	2 (22.2)	8 (53.3)	17 (65.4)	34 (55.7)
Decreasing food availability	4 (36.4)	2 (22.2)	4 (26.7)	6 (23.1)	16 (26.2)
Unintentional poisoning	0 (0)	1 (11.1)	1 (6.7)	0 (0)	2 (3.3)
Do not know	0 (0)	4 (44.4)	2 (13.3)	3 (11.5)	9 (14.8)

*Note*: The total number of respondents (*n*) is shown, along with the number of respondents expressed as a percentage of the total (%).

**TABLE A2:** Supporting informations (Qualitative statements when handing the picture of Hooded vulture to respondents and interviewer’s personal observations).

Qualitative statements highlighting the respondents’ cognisance of the decline of Hooded Vulture
*"It was here in former times"*
*"Where did this vulture go?"*
*"I have not seen it in our town/township for several years"*.
Qualitative statements highlighting the respondents’ cognisance of the causes of Hooded Vulture decline
*“Its body parts are used to treat several physical and mental diseases”*
*“Its body parts are used to bring good luck during gambling, competitions and contests.”*
*“Nigerians are very interested in this vulture (alive or dead)”*
Qualitative statements highlighting the low level of protection afforded to Hooded Vulture in Northern Benin
*“I can provide you with Hooded vultures if you really want them.”*
*“I had no idea that this bird was so precious to be protected by legislation.”*
Personal observations
*We saw traps for Hooded Vulture at three abattoirs: Banikoara, Firou, Péhonko*
*We saw hunter holding a shotgun, waiting for Hooded Vultures, at the abattoir of Nikki*
*We saw people at the abattoirs in Tanguiéta, Natitingou, Banikoara keeping organs that had been declared unsuitable for human consumption by veterinary services and which could have been offered as food to vultures*
