# Supplementary material for: Hooded Vultures Necrosyrtes monachus are at risk of extinction in Benin: A result of poaching for belief‐based use and decreasing food availability
Source: Ecol Evol. 2024 Apr 1;14(4):e11184. doi: 10.1002/ece3.11184 (PMC10985359; doi:10.1002/ece3.11184)
Supplement: Supplementary file 1 — Tables S1 [file ECE3-14-e11184-s001.docx]

**TABLE S1:** Supporting informations (Qualitative statements when handing the picture of Hooded vulture to respondents and interviewer’s personal observations)

| **Qualitative statements highlighting the respondents’ cognisance of the decline of Hooded Vulture** |
| --- |
| *"It was here in former times"* |
| *"Where did this vulture go?"* |
| *"I have not seen it in our town/township for several years".* |
| **Qualitative statements highlighting the respondents’ cognisance of the causes of Hooded Vulture decline** |
| *“Its body parts are used to treat several physical and mental diseases”* |
| *“Its body parts are used to bring good luck during gambling, competitions and contests.”* |
| *“Nigerians are very interested in this vulture (alive or dead)”* |
| **Qualitative statements highlighting the low level of protection afforded to Hooded Vulture in Northern Benin** |
| *“I can provide you with Hooded vultures if you really want them.”* |
| *“I had no idea that this bird was so precious to be protected by legislation.”* |
| **Personal observations** |
| *We saw traps for Hooded Vulture at three abattoirs: Banikoara, Firou, Péhonko* |
| *We saw hunter holding a shotgun, waiting for Hooded Vultures, at the abattoir of Nikki* |
| *We saw people at the abattoirs in Tanguiéta, Natitingou, Banikoara keeping organs that had been declared unsuitable for human consumption by veterinary services and which could have been offered as food to vultures* |
